# Modular hemipelvic endoprosthesis with a sacral hook: a finite element study

**DOI:** 10.1186/s13018-019-1338-z

**Published:** 2019-09-11

**Authors:** Bo Wang, Peidong Sun, Hao Yao, Jian Tu, Xianbiao Xie, Jun Ouyang, Jingnan Shen

**Affiliations:** 1 Department of Musculoskeletal Oncology, The First Affiliated Hospital of Sun Yat-Sen University, 58#, Zhongshan Road II, Guangzhou, 510080 Guangdong China; 2 Medical Biomechanical Key Laboratory of Guangdong Province, Department of Anatomy, Southern Medical University, Tonghe, Guangzhou, 510515 Guangdong China

**Keywords:** Pelvic tumor, Sacral hook, Hemipelvic prosthesis, Biomechanics, Finite element analysis

## Abstract

**Background:**

A novel hemipelvic endoprosthesis with a sacral hook was introduced previously, and its clinical outcome with midterm follow-up showed decreased prosthesis-related complications, especially decreased rate of aseptic loosening. The aim of present study was to evaluate the role of a sacral hook in prosthesis stability and the biomechanical properties of this hemipelvic endoprosthesis.

**Methods:**

A three-dimensional model of the postoperative pelvis was developed using computed tomography (CT) images. A force of 500 N was applied, and the distribution of stress and displacement was evaluated. Comparisons were performed to explore the role of the sacral hook in prosthesis stability. Prosthesis improvement was simulated to reduce unexpected breakage of the pubic connection plate.

**Results:**

In the reconstructed hemipelvis, stress distributions were concentrated on the superior area of the acetabulum, sacral connection component, and sacral hook. A maximum stress of 250 MPa was observed at the root of the sacral connection component. The sacral hook reduced the maximum stress and displacement by 14.1% and 32.5%, respectively, when the prosthesis was well fixed and by 10.0% and 42.1%, respectively, when aseptic loosening occurred. Increasing the thickness of the pubic connection plate from 2 to 3.5 mm reduced the maximum stress by 32.0% and 15.8%, respectively.

**Conclusion:**

A hemipelvic endoprosthesis with a sacral hook fulfills the biomechanical demands of the hemipelvis and is safe under static conditions. The sacral hook is important for prosthesis stability. Increasing the thickness of the pubic connection plate can reduce the maximum stress and risk of fatigue breakage.

## Background

Pelvic tumors, especially malignant tumors, greatly impact a patient’s survival and quality of life. Limb salvage surgery by prosthetic or biological reconstruction is favored over classical hemipelvectomy [1, 2]. However, reconstruction of pelvic bone defects remains challenging in musculoskeletal oncology. With improvements in chemotherapy and radiotherapy, the estimated 5-year survival rate for malignant pelvic tumors has reached 64.0 to 82.0% [1–4], and an increasing survival rate has led to a growing demand for limb function and extension of prosthesis life. Novel prosthesis designs and new fixation strategies have aimed to solve this problem, but they have had little impact according to the published literature.

We introduced a novel modular hemipelvic endoprosthesis with a sacral hook and evaluated its clinical effects by analyzing the midterm follow-up of 50 consecutive cases [4]. The sacral hook is a specialized part of the sacral connection component, designed to transform the great shearing force caused by the large angle between the sacral wing and the sacral connection component into compression force, and the aims of this design were to reinforce the prosthetic stability and to prolong prosthetic survival. The clinical results were promising regarding prosthesis-related complications and postoperative limb function. The prosthesis-related complication rate was 16.0%, with an estimated 3-year prosthesis survival of 89.0%, and the postoperative limb function reached 61.4% according to the Musculoskeletal Tumor Society (MSTS) scale [5]. Among those complications, aseptic loosening of the sacral connection component was reduced significantly, so we propose that the novel sacral hook plays an important role in prosthesis stabilization and survival, which must be tested and validated by biomechanical experiments. In addition, a relatively higher rate of pubic connection plate breakage due to unknown causes was discovered, which also should be explained and improved by a biomechanics study. Therefore, a comprehensive biomechanical study of the hemipelvic prosthesis with a sacral hook is necessary.

Finite element (FE) analysis is a method to computationally model reality in a mathematical form to better understand interactions within a highly complex system. It can accommodate large intersubject variations in bone geometry and material properties [6, 7]. An empirically validated FE model could provide information about the static and dynamic responses of joint structures under a variety of loading and boundary conditions that would be difficult or even impossible to obtain experimentally [8]. With all of these advantages, FE modeling has been widely used to analyze pelvic biomechanics [6, 7].

In this study, we performed FE analysis of the pelvis after reconstruction with a novel hemipelvic endoprosthesis with a sacral hook. The aims of this study were to (1) evaluate the biomechanical properties of the hemipelvic endoprosthesis, (2) explore the effects of the sacral hook under different situations, and (3) determine the causes of pubic connection plate breakage.

## Methods

### Endoprosthesis

**Fig. 1:**
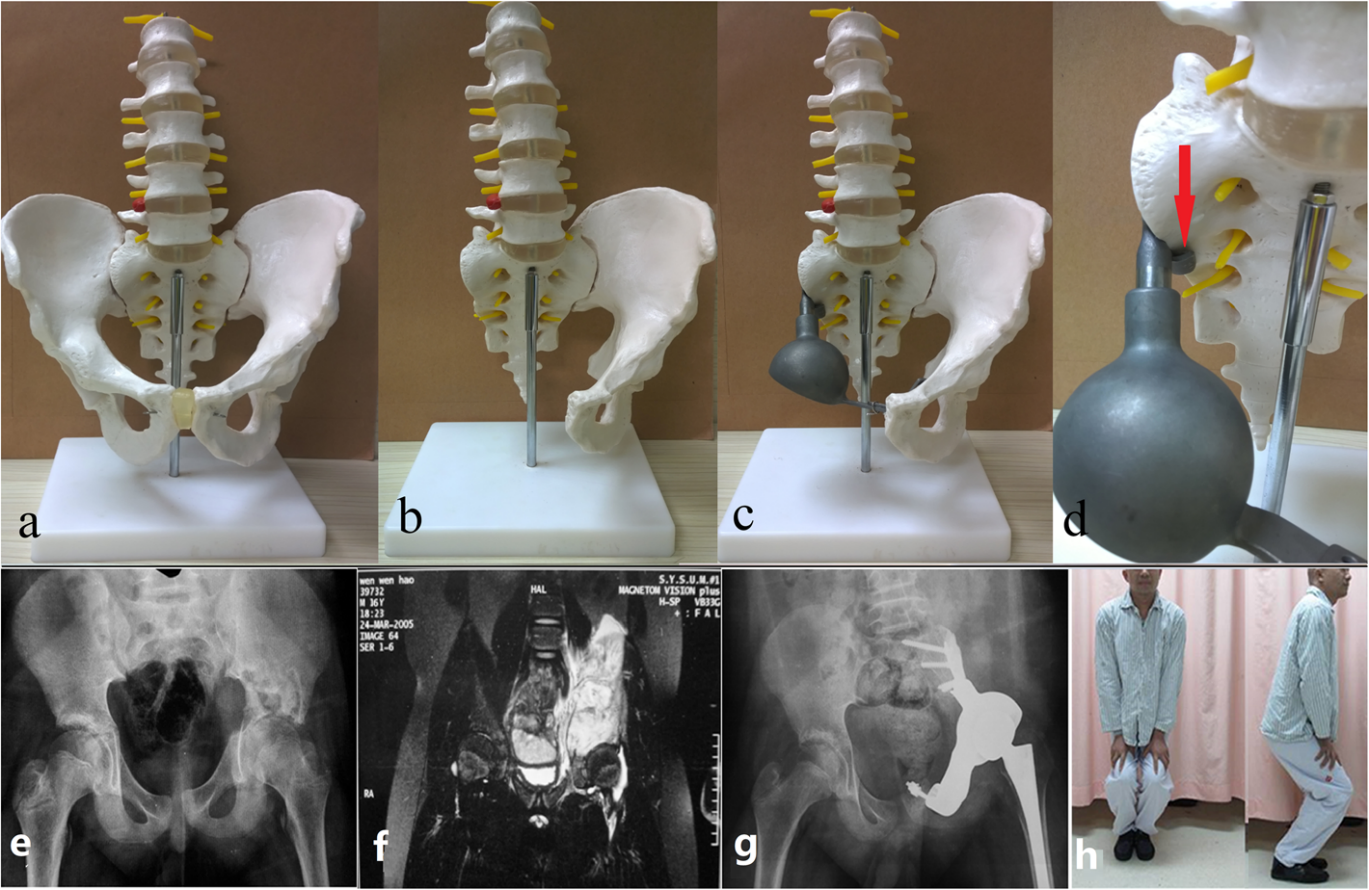
The hemipelvic endoprosthesis with a sacral hook used in our study. **a–d** Hemipelvic resection and reconstruction using a prosthesis with a sacral hook (red arrow). **e–h** The patient selected in this study had the best limb function and could walk without any support

The novel modular hemipelvic endoprosthesis with a sacral hook was designed by our research group, which was led by Prof. Shen (patent number: CN2017206233254). It was manufactured by Lidakang Science and Technology Co., Ltd. (Beijing, China) and is composed of a restrained total hip joint, a pubic connection plate, and a sacral connection component with a specifically designed sacral hook (Fig. 1). All components are made of a titanium alloy (TC4). The acetabulum component of the total hip joint was linked and fixed by the pubic connection plate to the pubis, and the sacral connection component was fixed to the sacrum; the sacral hook was inserted and hooked to the fornix of the sacrum (Fig. 1). Vancomycin-containing bone cement was used to seal and reinforce the prosthesis and prevent infections.

The surgical procedure was similar to that previously reported [3]; the gluteus medius and minimus were usually sacrificed to achieve a safe surgical margin. The rotation center of the hip joint was slightly shifted superomedially to reduce the dead cavity, improve soft tissue coverage, and theoretically reduce the deep infection rate despite aggravation of the gluteus weaknesses, which can worsen functional performance. After 8 to 10 weeks of bed rest to allow scar tissue formation and construction stabilization, increased weight bearing was allowed, with the aid of crutches.

### Patient demographics

The patient demographics were the same as previously reported [4]. Fifty consecutive patients (29 males and 21 females; average age, 26 years; range, 12 to 67 years) who underwent reconstructive surgery with the modular hemipelvic endoprosthesis after tumor resection at the Musculoskeletal Oncology Center of the First Affiliated Hospital of Sun Yat-Sen University between 2003 and 2013 were enrolled. Pathological diagnoses were confirmed via preoperative core needle biopsy and included 21 osteosarcomas, 11 Ewing’s sarcomas, 8 chondrosarcomas, 5 metastatic malignancies, 3 fibrosarcomas, and 2 giant cell tumors of the bone. Neoadjuvant or adjuvant treatments, such as chemotherapy or radiotherapy, were applied according to their indications. Before surgery, written informed consent was obtained from all patients or from the parents of patients younger than 18 years. All patients underwent tumor resection and reconstruction surgery with the novel hemipelvic endoprosthesis and were followed routinely.

We chose one typical patient (male, 34 years old, 178 cm in height, 74 kg in weight) diagnosed with Enneking stage IIB [9] osteosarcoma to be the subject of FE analysis. He underwent an Enneking type [10] I, II, and III resection with wide surgical margins achieved by sacrificing the gluteus minimus and part of the gluteus medius. He exhibited the best limb function among all patients and could walk without any support (Fig. 1). His postoperative images showed that the reconstruction was representative, besides the material properties used in FE model reconstruction were statistically measured, so we believed that the FE analysis of this typical patient could be sufficient. He provided additional written informed consent to publish the details of his case. This study was approved by the ethics committee of the First Affiliated Hospital of Sun Yat-Sen University.

### Three-dimensional model of the reconstructed pelvis

A three-dimensional model of the reconstructed pelvis was developed from the chosen patient at 12 months postoperatively. The patient underwent a long-axis computed tomography (CT) scan from the third lumbar vertebra to the middle femur, while both lower extremities were kept in a neutral position (Philips Brilliance 64CT, Philips Healthcare, the Netherlands; slice thickness = 0.5 mm, 1614 slices). Metal artifacts were eliminated using the difference in the gray value between the prosthesis and artifacts on the CT image. The image data were then input into Mimics (version 14.01, Materialise, Belgium) and Geomagic Studio (version 2012, Geomagic, USA) software to build the three-dimensional reconstructed model of the postoperative pelvis and L4 and L5. Because the patient’s normal pelvis could not be reconstructed directly, we modeled the normal pelvis through mirror imaging of the unaffected side. Muscles and ligaments were not reconstructed because of their minor effects on weight bearing after wide resection [11]. We built the L4/5 and L5/S1 intervertebral disks according to the report by Schmidt et al. [12].

### FE model of the pelvis

The three-dimensional reconstructed models of the normal and postoperative pelvis were input into Abaqus software (version 6.10, Dassault Systemes, France), and mesh refinement tests were performed. The element type and mesh number of the final FE models are shown in Table 1. We applied a force of 500 N (2/3 of body weight) to the lamina terminalis of the fourth lumbar vertebra along the longitudinal axis of the normal and postoperative pelvis to simulate standing on 2 ft. The material properties of the prosthesis were determined by Lidakang Science and Technology Co., Ltd. (Beijing, China). All prosthetic components were made of continuous isotropic elastomeric materials. The detailed properties [6, 12–15] are listed in Table 2.

**Table 1:** Figures of FE model

	Normal pelvis	Postoperative pelvis	Prosthesis
Mesh type	C3D4	C3D4, D3D10M	C3D10M
Node	55,417	222,897	145,621
Mesh	238,375	372,543	172,144

**Table 2:** Material properties

Material	Elastic modulus	Poisson’s ratio	Yield strength
Cortical bone	17 GPa	0.3	120 MPa
Cancellous bone	800 MPa	0.3	
Titanium alloy TC4	115 GPa	0.33	905 MPa
Nucleus pulposus	0.9 MPa	0.5	
Fibrous ring	1.35 MPa	0.4	

To evaluate the effectiveness of the sacral hook, we simulated and compared the stress and displacement distribution of the prosthesis with and without the sacral hook under different conditions, such as fixation and aseptic loosening of the screws to the sacral wing. We measured the load on the sacral hook in these situations and simulated possible prosthetic improvement to the pubic connection plate by increasing its thickness.

## Results

### FE model of the normal pelvis

The stress distribution and displacement of the normal pelvis were similar to those previously reported [11]. Stress was concentrated on the superior area of the acetabulum, arcuate line, superior area of the greater sciatic notch, sacroiliac joint, and sacral midline while standing on 2 ft. A maximum stress of 1.63 MPa was present at the superior area of the greater sciatic notch while standing on 2 ft. Displacement was distributed along the iliac crest and the lateral edge of the acetabulum, and a maximum displacement of 0.02 mm was observed around the obturator foramen.

### FE model of the postoperative pelvis

**Fig. 2:**
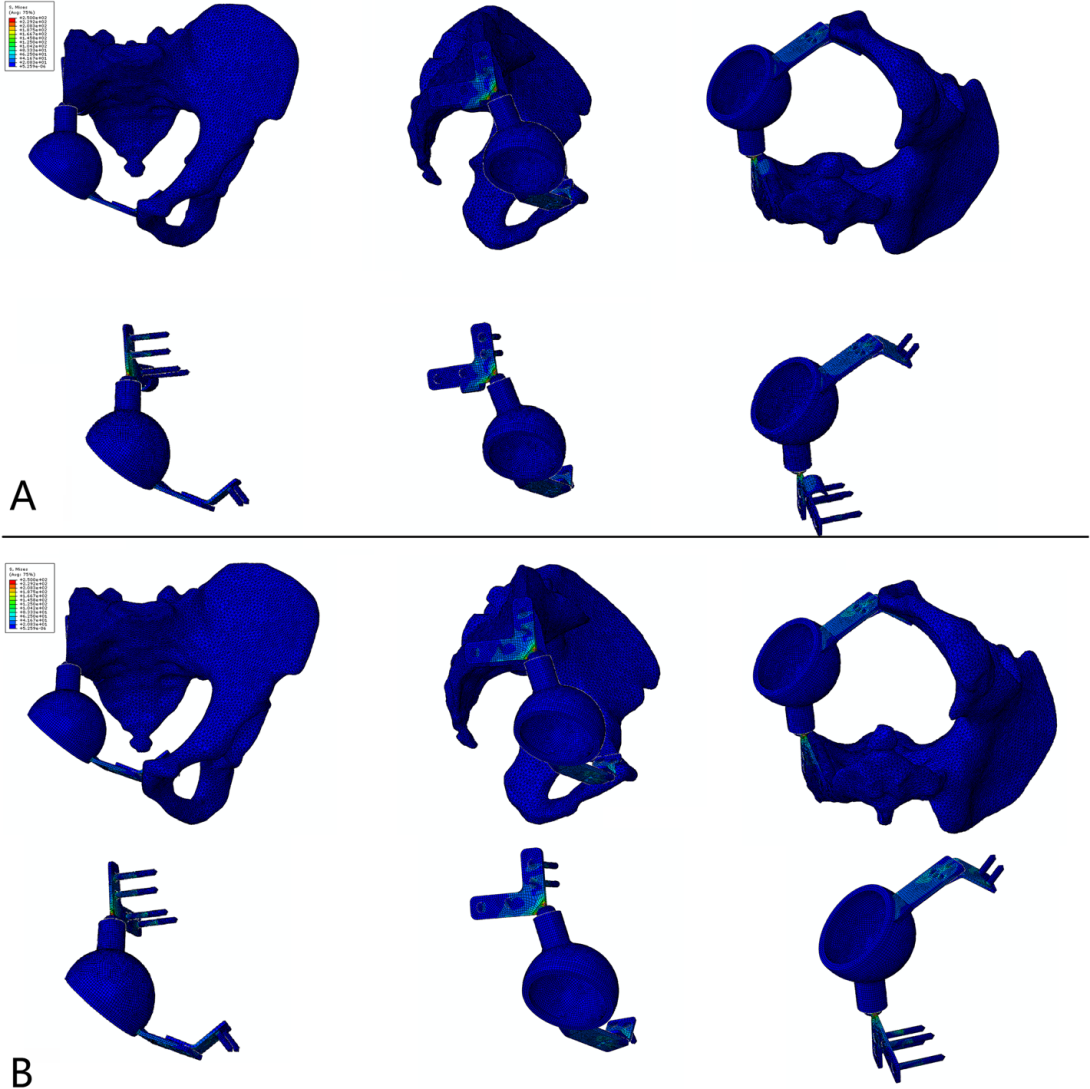
Stress distribution of the pelvis reconstructed using a prosthesis with a sacral hook under different conditions. **a** Stress distribution when the prosthesis was well fixed, max = 250 MPa. **b** Stress distribution when aseptic loosening of the sacral connection component occurred, max = 279 MPa

The stress and displacement distribution of the unaffected side were similar to those of the normal pelvis in the standing position. For the reconstructed side, stress concentrations were observed in the superior area of the acetabulum, sacral connection component, sacral hook, and pubic connection plate in the standing position. A maximum stress of 250 MPa was observed at the root of the sacral connection component, while the maximum stress of the pubic connection plate reached 125 MPa (Fig. 2). Displacement of the reconstructed side was mainly concentrated along the lateral edge of the acetabulum, with a maximum value of 0.56 mm.

### Effectiveness of the sacral hook under different situations

**Fig. 3:**
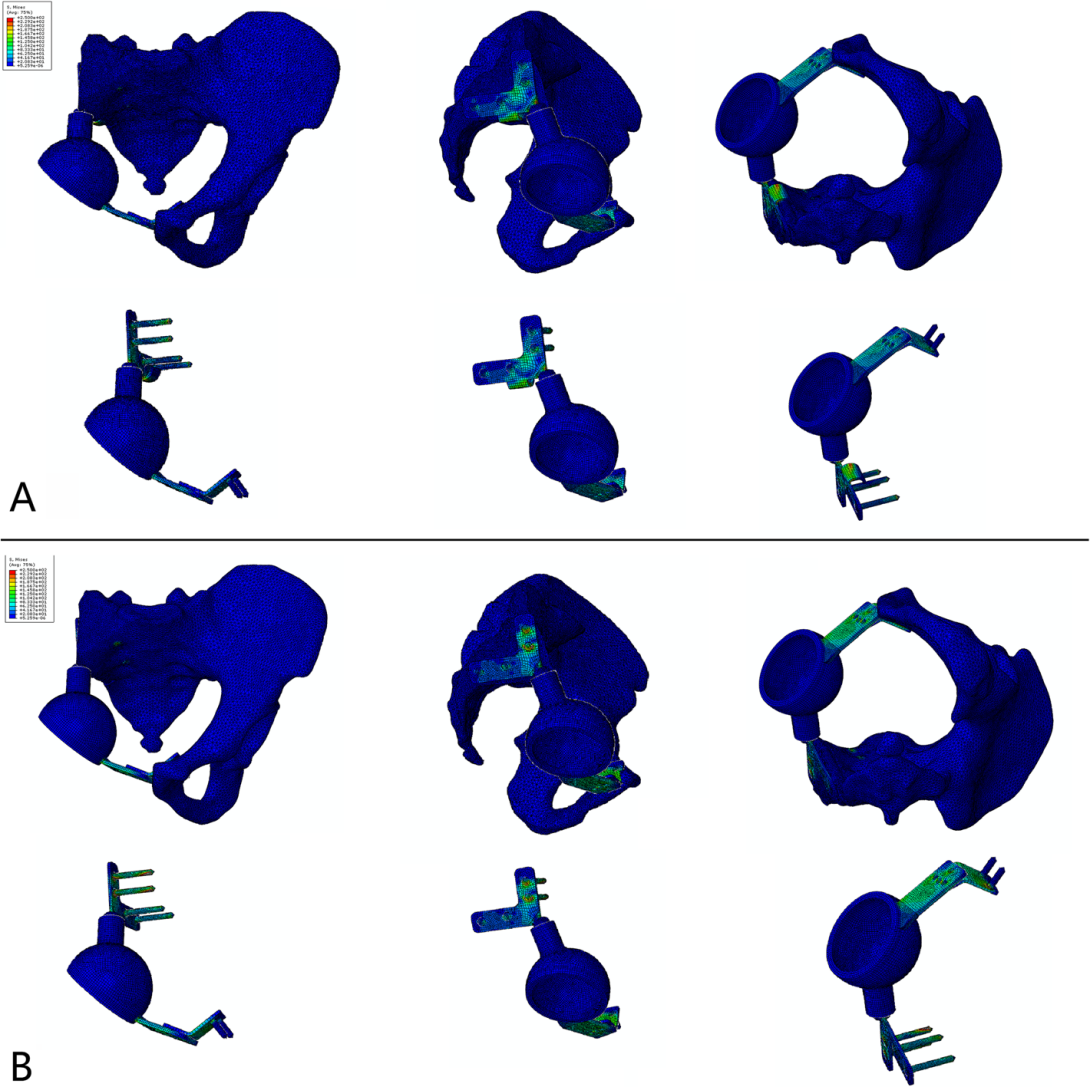
Stress distribution of the pelvis reconstructed using a prosthesis without a sacral hook under different conditions. **a** Stress distribution when the prosthesis was well fixed, max = 291 MPa. **b** Stress distribution when aseptic loosening of the sacral connection component occurred, max = 310 MPa

**Fig. 4:**
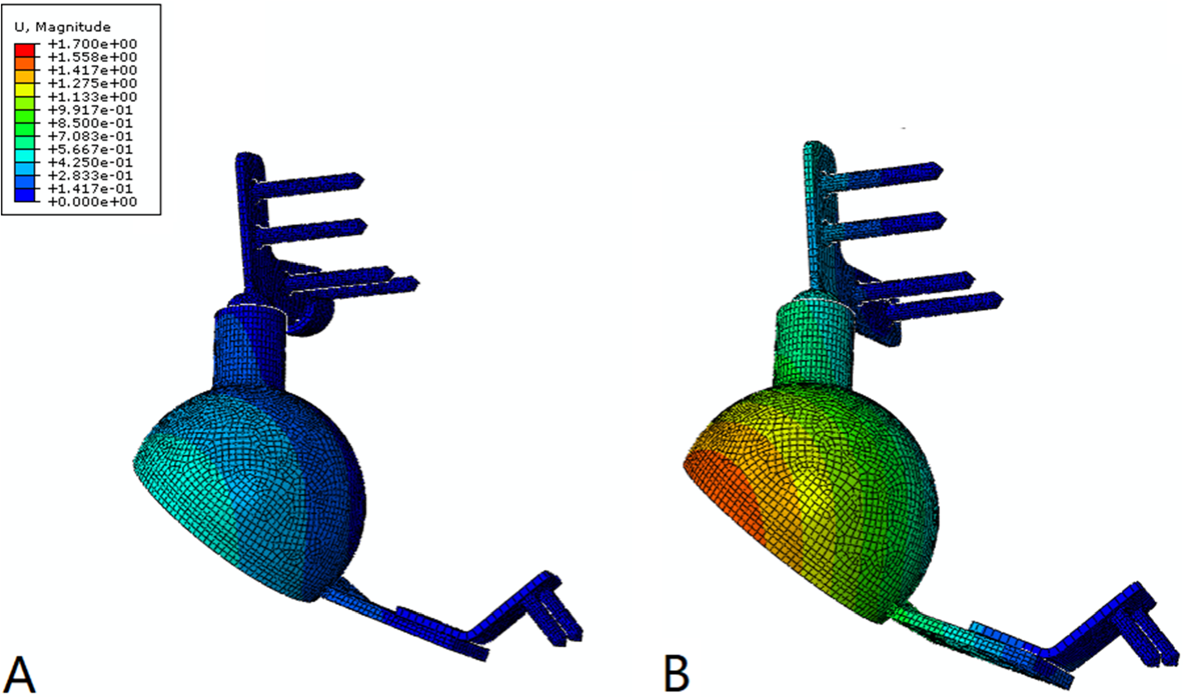
Comparison of displacement distributions for prostheses with and without a sacral hook. **a** Prosthesis with a sacral hook, maximum displacement = 0.56 mm. **b** Prosthesis without a sacral hook, maximum displacement = 1.52 mm

**Fig. 5:**
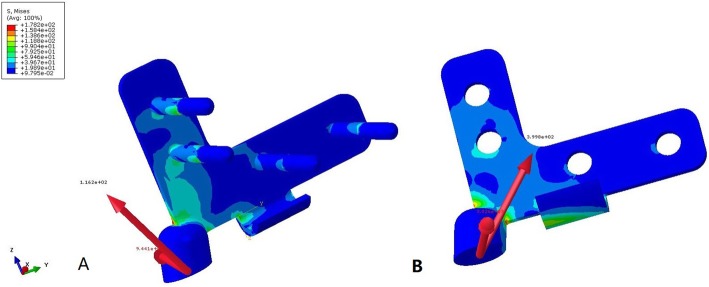
Comparison of the load borne by the sacral hook under different conditions. **a** When the prosthesis was well fixed, the maximum load = 116 N. **b** When aseptic loosening of the sacral connection component occurred, the maximum load = 399 N

The stress and displacement distribution patterns were similar under different conditions. For the prosthesis without the sacral hook, the maximum stress was 291 MPa when the sacral screws were well fixed and increased to 310 MPa when the screws were loosened. The maximum displacements in these two situations were 0.83 mm and 1.52 mm, respectively. For the prosthesis with the sacral hook, the maximum stress was 250 MPa (14.1% reduced) when the sacral screws were well fixed and 279 MPa (10.0% reduced) when the screws were loosened (Figs. 2 and 3). The maximum displacements in these two situations were 0.56 mm (32.5% reduced) and 0.88 mm (42.1% reduced), respectively (Fig. 4). The load on the sacral hook was 116 N (23.2%) when the screws were well fixed and 399 N (79.8%) when the screws were loosened (Fig. 5).

### Prosthesis improvement

**Fig. 6:**
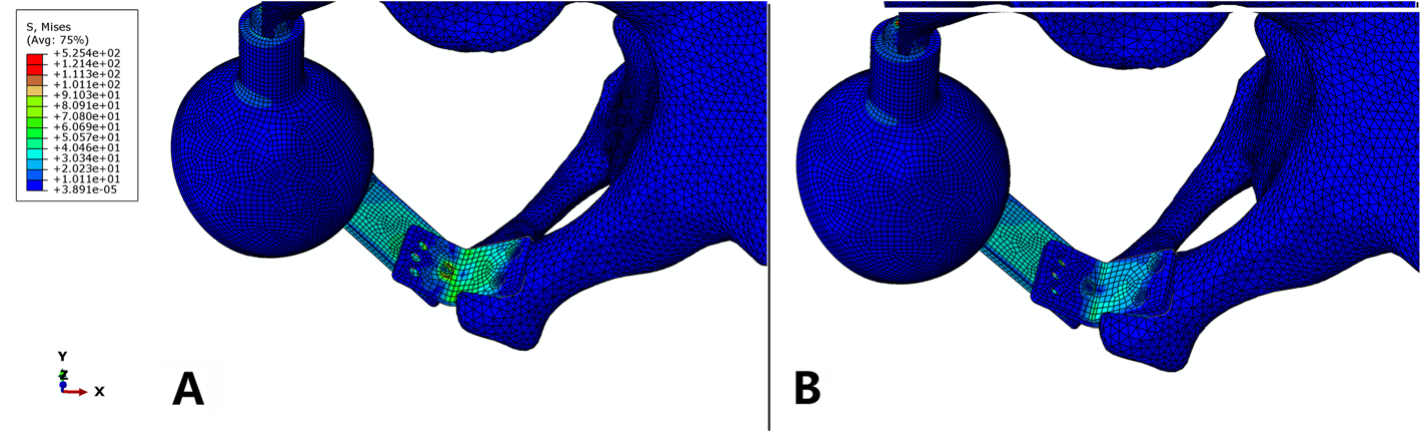
Simulation of prosthesis improvement by increasing the thickness of the pubic connection plate. **a** Thickness = 2 mm, maximum stress = 125 MPa. **b** Thickness = 3.5 mm, maximum stress = 85 MPa

The pubic connection plate was made of the TC4 titanium alloy, the properties of which are not easy to change or improve; therefore, we increased the plate thickness to reduce the concentrated stress. The thickness of the pubic connection plate was 2 mm; the maximum stress was 125 MPa when the prosthesis was well fixed and 166 MPa when the sacral screws were loosened. We increased the thickness to 3.5 mm after simulation and FE analysis; the maximum stress of the pubic connection plate decreased to 85 MPa (32.0% reduced) when the prosthesis was well fixed and 140 MPa (15.8% reduced) when the sacral screws were loosened (Fig. 6).

## Discussion

### Endoprosthesis

Limb salvage surgery, combined with chemotherapy and radiotherapy, has replaced traditional hemipelvectomy in treating pelvic tumors due to the similar survival and recurrence rates [13–15]. Greater improvement in quality of life and decreased psychological trauma and physical disability renders the procedure favorable and acceptable. Reconstruction with a hemipelvic endoprosthesis is the most common choice due to its reliability and effectiveness. However, the high complication rate and insufficient follow-up data make this nonbiological reconstruction strategy controversial. Hence, we have designed a hemipelvic endoprosthesis with a novel sacral hook. The sacral hook was designed to transform the great shearing force of the sacral fixation screws into compression stress when installed on the dome-shaped anterior side of sacrum; thus, it can share the load of the sacral connection component screws and increase the stability and reliability of the prosthesis. The preliminary clinical results were promising regarding postoperative limb function and prosthesis-related complications, especially regarding the low rate of aseptic loosening of the sacral connection component. However, breakage of the pubic connection plate due to unknown causes was more common than expected; therefore, we aimed to fully explore the biomechanical properties of the novel prosthesis and the role and mechanism of the sacral hook in the stability of the reconstruction system.

It is difficult to assess the stress and displacement distributions throughout the entire pelvis using simplified mathematical models, implanted prostheses, or via experiments with cadaveric tissue due to its complex biomechanics [13], especially for pelvises reconstructed with nonstandard prostheses. However, FE analysis, which is a method to computationally model reality in a mathematical form to better understand the interactions within a highly complex system, provides an attractive approach for analyzing pelvic mechanics. The FE model was built directly from CT images of the selected patient, which are similar to the actual conditions, and simulation of prosthesis improvement using FE analysis was convenient.

### Biomechanical properties

Approximately two thirds of the body weight is borne by the pelvis under physiological conditions. In our study, a load of 500 N was applied, which would be sufficient for adult patients. The stress distributions of the normal pelvis when standing on 2 ft were identical to those previously reported [16], which validated our FE model. The maximum stress of 1.63 MPa observed was less than the yield strength of the pelvic cortex [16], and a maximum displacement of 0.02 mm was observed around the obturator foramen, which indicated the stability and reliability of the normal pelvis under physiological conditions.

The stress and displacement distribution of the unaffected side of the postoperative pelvis were similar to that of the normal pelvis, and these findings indicated that the prosthetic reconstruction of the affected side fulfilled its biomechanical requirements, with only a small influence on the stress and displacement distribution of the unaffected side.

The stress distributions of the postoperative pelvis were concentrated mostly on the superior area of the acetabulum, sacral connection component, sacral hook, and pubic connection plate in the standing position. The peak stress (250 MPa) was observed at the root of the sacral connection component. The maximum stress of the pubic connection plate reached 125 MPa. However, both distributions were much lower than the tensile strength and yield strength of the TC4 titanium alloy. A maximum displacement of 0.56 mm was observed at the lateral edge of the artificial acetabulum. The acceptable stress and displacement suggested that the prosthesis was stable and reliable under static conditions.

### Role of the sacral hook in prosthesis stability

Achieving the goal of the specialized design, the sacral hook reduced the maximum stress and displacement by 10.4% and 36.4%, respectively, when the prosthesis was well fixed. This effect became more obvious when the fixation screws to the sacrum were loosened, and the maximum stress and displacement were reduced by 6.1% and 45.4%, respectively. These results validated the role of the sacral hook in improving prosthesis stability and reliability, especially when aseptic loosening of the sacral connection component occurred. This sacral hook helped to share the load of the sacral connection component. Under normal conditions, it bore 23.2% (116 N) of the load, which increased to 79.8% (399 N) when aseptic loosening occurred. Therefore, the sacral hook could protect the prosthesis, especially the sacral connection component, from aseptic loosening, which may explain the relatively low rate of aseptic loosening during clinical follow-up.

Based on these promising biomechanical results, using the sacral hook with this hemipelvic prosthesis is safe, and fatigue is unlikely to occur under static conditions. However, the stresses incurred during static positions are not the largest stresses borne by the pelvis [17]. Stress borne by the pelvis increases dramatically from four to seven times the body weight when walking steadily and is even more than ten times the body weight when running and jumping [18, 19]. The safety of the prosthesis must be guaranteed under dynamic conditions because the intention of limb salvage surgeries is improved limb function; therefore, the biomechanical properties of the prosthesis under loaded gait cycles should be investigated in the future. In contrast, improvements were required to reduce the peak stress, especially when unexpected breakage of the pubic connection plate occurred during clinical follow-up.

### Simulated prosthesis improvement

Although the maximum stresses of the pubic connection plate under different conditions were much less than the tensile strength and yield strength of the TC4 titanium alloy, breakage occurred more often than expected. Ji et al. [20] reported the concentration of stress on the pubic connection plate under a gait cycle and found a maximum stress of 280 MPa. We suggest that repeated periodic stress under a gait cycle may cause fatigue breakage. Improvement of the prosthesis to reduce the concentration of stress on the pubic connection plate was necessary.

The maximum stresses were reduced by 32.0% and 15.8% under well-fixed conditions and aseptic loosening, respectively, after increasing the thickness of the plate from 2 to 3.5 mm. We propose that a thickness of 3.5 mm is a good balance between stress reduction and prosthesis assembling, with screw fixation and soft tissue coverage. The new 3.5-mm-thick pubic connection plate is currently being used in surgeries, and its clinical effects must be validated in the future.

This FE study had several limitations. First, the FE model was based on CT images of a single patient. Second, muscles and ligaments were not simulated. Third, biomechanics under dynamic conditions were not investigated. We hope to address these limitations in a future study.

All these findings have been translated into clinical practice to decrease complications and increase life qualities of patients with pelvic tumor. What is more, finite element model reconstructed in this study could be used in preimplantation validation of three-dimensional printed prosthesis developed from the same CT images for the same patient. We are working to establish a standard procedure of guided tumor resection and validated three-dimensional printed prosthesis, all these steps are based on image reconstruction and finite element analysis, and the aim is to decrease disease recurrence and increase prosthesis stability.

## Conclusions

The hemipelvic endoprosthesis with a sacral hook fulfilled the physiological and biomechanical demands of the hemipelvis. The endoprosthesis was safe under static conditions. The sacral hook played an important role in prosthesis stability. Increasing the thickness of the pubic connection plate could reduce the maximum stress and fatigue breakage.

## Data Availability

The datasets generated and/or analyzed during the current study are not publicly available due to huge file size but are available from the corresponding author on reasonable request.

## Abbreviations

CT: Computed tomography
FE: Finite element
MSTS: Musculoskeletal Tumor Society
